# Identification of G-quadruplex clusters by high-throughput sequencing of whole-genome amplified products with a G-quadruplex ligand

**DOI:** 10.1038/s41598-018-21514-7

**Published:** 2018-02-15

**Authors:** Wataru Yoshida, Hiroki Saikyo, Kazuhiko Nakabayashi, Hitomi Yoshioka, Daniyah Habiballah Bay, Keisuke Iida, Tomoko Kawai, Kenichiro Hata, Kazunori Ikebukuro, Kazuo Nagasawa, Isao Karube

**Affiliations:** 1 School of Bioscience and Biotechnology, Tokyo University of Technology, 1404-1 Katakura-machi, Hachioji, Tokyo, 192-0982 Japan; 2 Department of Maternal-Fetal Biology, National Research Institute for Child Health and Development, 2-10-1 Ookura, Setagaya, Tokyo, 157-0074 Japan; 3 Biology Department, Umm Al-Qura University, P.O. Box 715, Makkah, 21955 Saudi Arabia; 4 Molecular Chirality Research Center, Synthetic Organic Chemistry, Department of Chemistry, Graduate School of Science, Chiba University, 1-33 Yayoi, Inage, Chiba, 263-8522 Japan; 5 Department of Biotechnology and Life Science, Tokyo University of Agriculture and Technology, 2-24-16 Naka-cho, Koganei, Tokyo, 184-8588 Japan

## Abstract

G-quadruplex (G4) is a DNA secondary structure that has been found to play regulatory roles in the genome. The identification of G4-forming sequences is important to study the specific structure-function relationships of such regions. In the present study, we developed a method for identification of G4 clusters on genomic DNA by high-throughput sequencing of genomic DNA amplified via whole-genome amplification (WGA) in the presence of a G4 ligand. The G4 ligand specifically bound to G4 structures on genomic DNA; thus, DNA polymerase was arrested on the G4 structures stabilised by G4 ligand. We utilised the telomestatin derivative L1H1-7OTD as a G4 ligand and demonstrated that the efficiency of amplification of the G4 cluster regions was lower than that of the non-G4-forming regions. By high-throughput sequencing of the WGA products, 9,651 G4 clusters were identified on human genomic DNA. Among these clusters, 3,766 G4 clusters contained at least one transcriptional start site, suggesting that genes are regulated by G4 clusters rather than by one G4 structure.

## Introduction

G-quadruplex (G4) is a DNA secondary structure composed of two or more stacking G-quartets, a planar array of four guanine bases connected by a Hoogsteen hydrogen bond, and stabilised by a monovalent cation^1^. In genomic DNA, G4-forming sequences were first described in immunoglobulin switch regions and telomeric DNA at the ends of chromosomes^2,3^ and have since been identified in several regulatory regions, such as transcription factor binding sites and promoters^4–6^. In promoter regions, G4-forming sequences are involved in transcriptionally activating^7–9^ or silencing gene expression^10,11^. In addition, G4s have been reported to be involved in replication^12^, DNA recombination^13^ and splicing processes^14^.

Identification of G4-forming sequences in the genome is necessary to elucidate the biological functions of G4. *In silico* analysis has revealed that the putative G-quadruplex forming sequences (PQS) are enriched in promoters, CpG islands, 5′UTRs, first exons, first exon/intron junctions and nuclease-hypersensitive sites^15–17^. Furthermore, putative duplex-derived interstrand G4-forming sequences have been identified^18,19^. However, the use of computational analysis alone is not sufficient to identify G4 regions precisely. We previously identified 1998 G4-forming sequences in mouse CpG islands using fluorescent-labelled G4 ligand with a mouse CpG island microarray^20,21^. Clusters of G4-forming sequences that promote transcription and replication-dependent DNA damage induced by a G4 ligand have been identified in oncogenes and tumour-suppressor genes by ChIP-Seq of the DNA damage marker γH2AX^22^. Recently, 716,310 G4-forming sequences stabilised by G4 ligand pyridostatin (PDS) and 525,890 G4-forming sequences stabilised by K^+^ were identified in the human genome by combining polymerase stop assay with Illumina next-generation sequencing (G4-seq)^23^.

In this study, we performed a whole genome amplification (WGA)^24^ in the presence of a G4 ligand, followed by high-throughput sequencing of WGA products to identify G4 clusters in human genomic DNA. It has been reported that DNA polymerase was arrested on G4 structures stabilised by G4 ligand^25^. This led us to hypothesise that genomic DNA would be amplified by WGA, except for G4 clusters, in the presence of a G4 ligand. Hence, we could identify G4 clusters by analysing the WGA products using high-throughput sequencing technologies.

## Results

### Analysis of inhibitory activity of G4 ligand against DNA polymerase extension on G4-forming sequences

**Figure 1:**
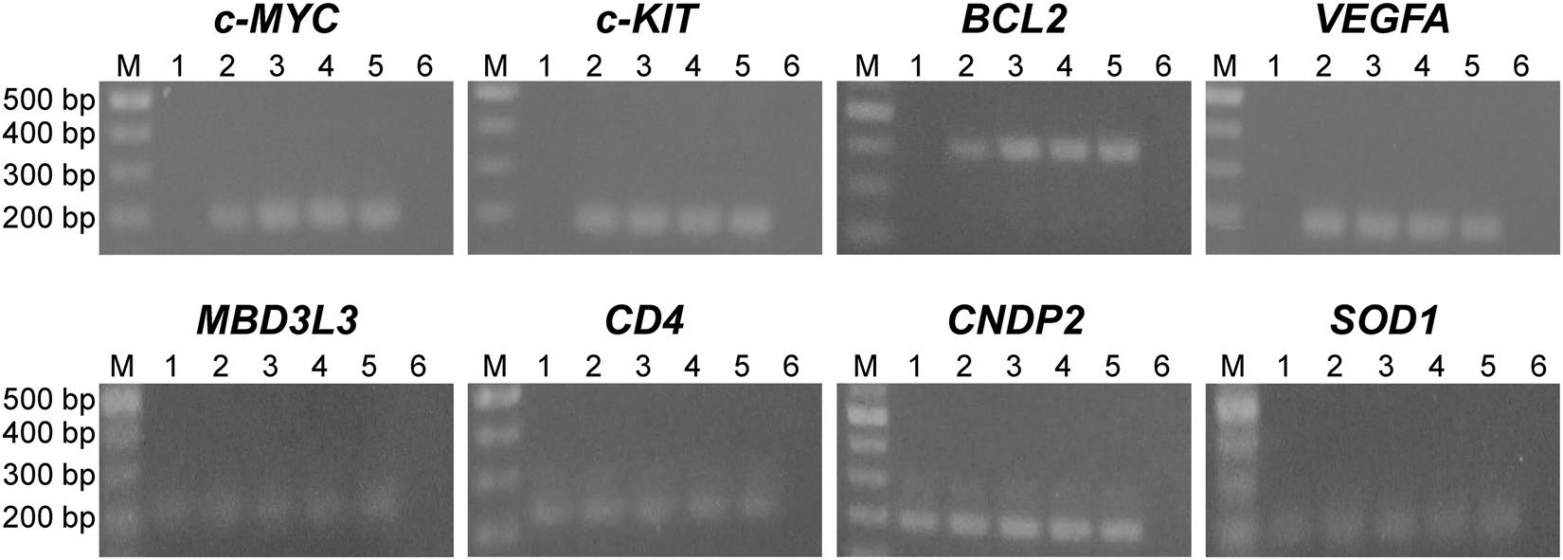
PCR stop assay. G4-forming regions [*c-MYC* (191 bp), *c-KIT* (192 bp), *BCL2* (398 bp) and *VEGFA* (192 bp)] and non-G4-forming regions [*MBD3L3* (213 bp), *CD4* (222 bp), *CNDP2* (194 bp), and *SOD1* (207 bp)] were amplified from 100 ng of HeLa genomic DNA by PCR in the presence of 1000 nM (lane 1), 100 nM (lane 2), 10 nM (lane 3), 1.0 nM (lane 4) or the absence of 7OTD (lane 5). As a control, PCR was performed without template DNA (lane 6). Lane M contains DNA markers. PCR products were analysed on 2% agarose gel. The cropped gels are used in the figure, and full-length gels are shown in Fig. S1.

The G4 ligand 7OTD is a telomestatin derivative that binds to the top of the G-tetrad structure through π-stacking and electrostatic interactions^26–28^. To investigate whether 7OTD inhibits DNA polymerase extension on G4-forming regions, polymerase chain reaction (PCR) was performed on the human genomic DNA in the presence of 7OTD. For amplifying G4-forming regions, PCR primers for *c-MYC*, *c-KIT*, *BCL2* and *VEGFA* G4 regions in the human genomic DNA were designed. For amplifying non-G4-forming regions, PCR primers for *MBD3L3*, *CD4*, *CNDP2*, and *SOD1* were designed. In the absence of 7OTD, all of these regions were accurately amplified from genomic DNA by PCR (Fig. 1). The G4-forming and non-G4-forming regions were amplified in the presence of less than 100 nM 7OTD. In the presence of 1 µM 7OTD, the non-G4-forming regions were amplified, whereas the G4-forming ones were not. These results demonstrate that 7OTD specifically inhibits DNA polymerase extension on G4-forming regions in PCR.

### Measurement of removal efficiency of BODIPY-labelled 7OTD from genomic DNA

PCR is a suitable method to confirm the efficiency of WGA of target regions; however, owing to the interference of 7OTD with PCR in the G4-forming regions, 7OTD should be removed from WGA products before PCR analysis. To evaluate the removal efficiency of 7OTD from genomic DNA, 10 µM BODIPY-labelled 7OTD was incubated with human genomic DNA. Then, LiCl solution (final concentration 4 M) was added to the mixture since lithium ions destabilise G4 structures^29^. The genomic DNA was purified by gel filtration and then the fluorescence intensity of BODIPY was measured to calculate the efficiency of removal of the residual BODIPY-labelled 7OTD from the genomic DNA. The results showed that 4.5% of the fluorescence intensity was detected in the purified samples, indicating that more than 95% of BODIPY-labelled 7OTD was successfully removed from genomic DNA by LiCl and gel filtration.

**Figure 2:**
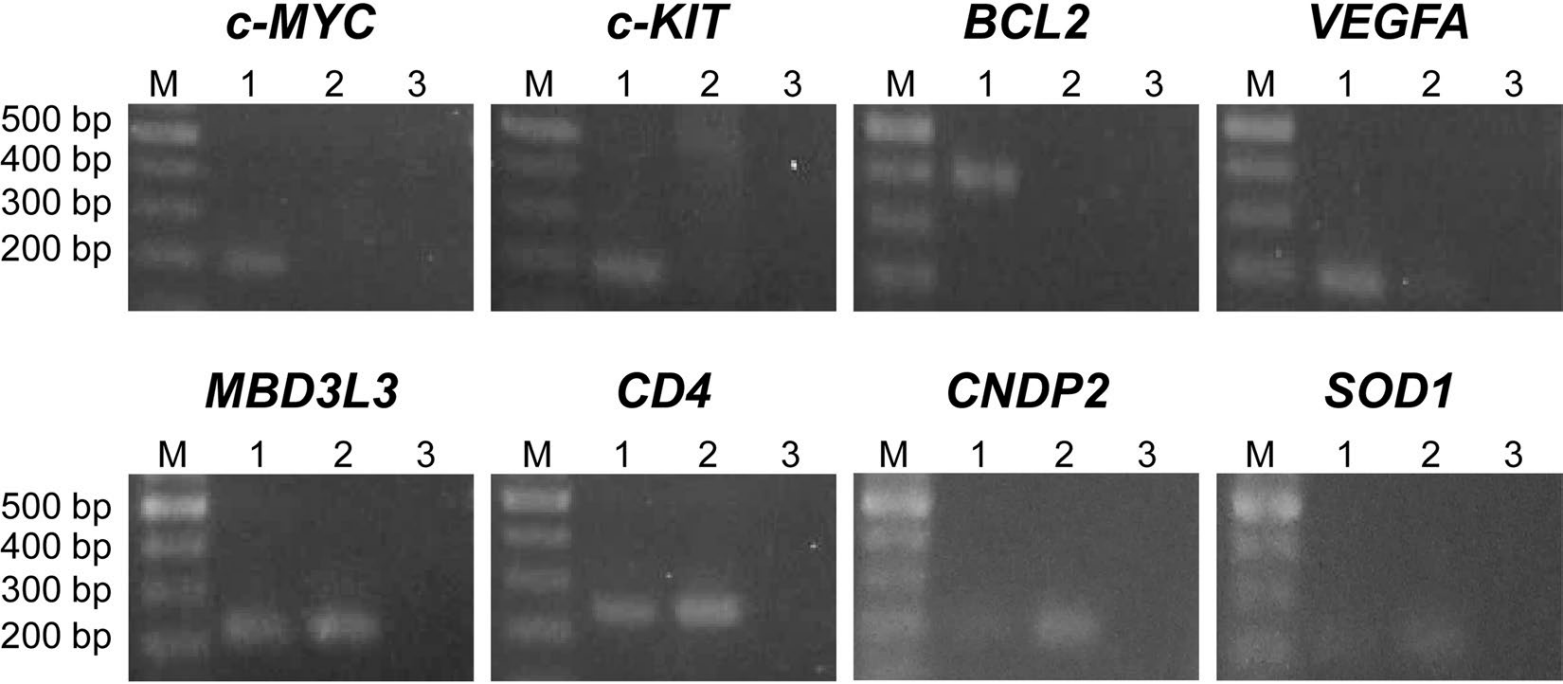
Analysis of the effect of residual 7OTD on PCR. PCR was performed using genomic DNA purified from a mixture of 1 µM 7OTD and genomic DNA (lane 1). As a control, PCR was performed using a mixture of genomic DNA and 7OTD as a template (lane 2) or without any template DNA (lane 3). Lane M contains DNA markers. The cropped gels are used in the figure, and full-length gels are shown in Fig. S2.

In the PCR analysis, 1 µM 7OTD inhibited PCR on G4-forming regions, but 100 nM 7OTD did not interfere with the reaction. Therefore, we assumed that 1 µM 7OTD is a suitable concentration for the WGA reaction, since the amount of 7OTD remaining after the purification would be less than 50 nM, which would not subsequently inhibit the PCR. To confirm that the residual 7OTD would not inhibit PCR on G4-forming regions, PCR was performed using genomic DNA purified from a mixture of 1 µM 7OTD and genomic DNA. The results showed that no PCR inhibition for *c-MYC*, c*-KIT*, *BCL2* and *VEGFA* G4 regions was detected (Fig. 2).

### Whole genome amplification in the presence of 7OTD

**Figure 3:**
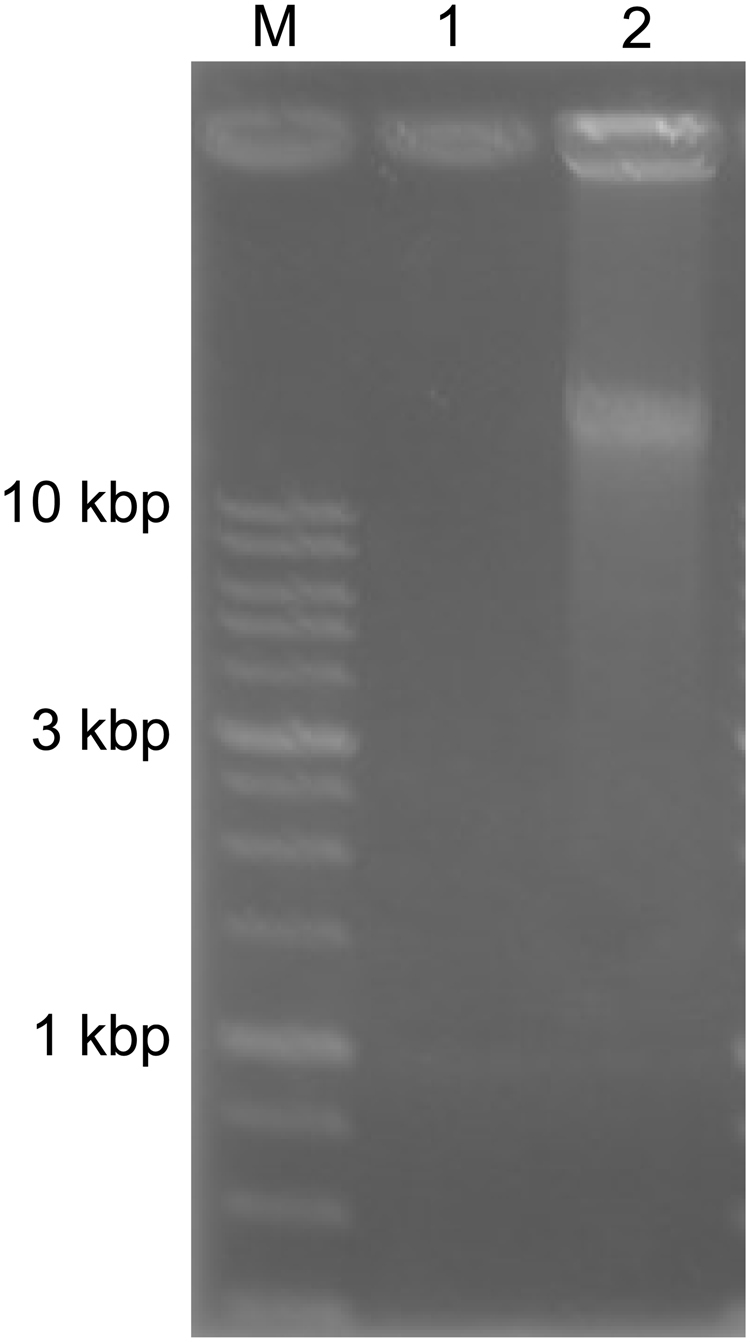
Agarose gel analysis of the WGA products. WGA was performed in the presence of 1 µM 7OTD (lane 1) or the absence of 7OTD (lane 2). Lane M contains DNA markers. The full-length gel is shown in Fig. S3.

WGA based on multiple displacement amplification using Phi29 DNA polymerase was utilised. In this system, the average product length is typically greater than 10-kb. When HeLa genomic DNA was amplified by WGA in the absence of 7OTD, WGA products of around 23-kb were obtained (Fig. 3). On the other hand, when HeLa genomic DNA was amplified by the WGA in the presence of 1 µM 7OTD, WGA products were not detected in 1% agarose gel electrophoresis. The G4-seq analysis revealed that the human genome contains 716,310 G4-forming sequences stabilised by a G4 ligand^23^. These results suggest that Phi29 DNA polymerase would be arrested on numerous G4-forming regions on genomic DNA and its inhibition would reduce the yield of total WGA products.

**Figure 4:**
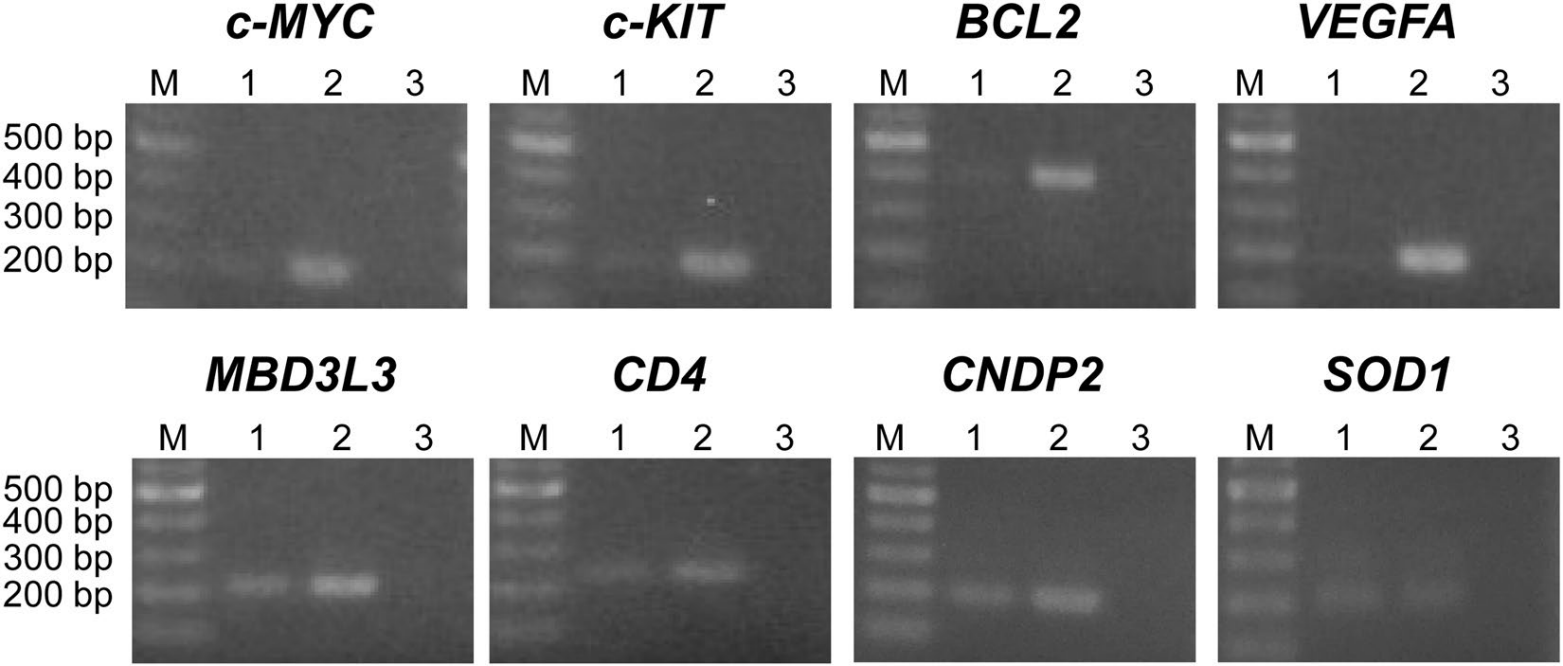
PCR analysis of the WGA products. PCR was performed using the products amplified by WGA in the presence (lane 1) or absence of 7OTD (lane 2). As a control, PCR was performed without any template DNA (lane 3). Lane M contains DNA markers. The cropped gels are used in the figure, and full-length gels are shown in Fig. S4.

To analyse whether 7OTD specifically inhibits the extension of DNA polymerase on G4-forming regions in WGA, the WGA products were purified by LiCl and gel filtration and analysed by PCR. After gel filtration of the products amplified by WGA in the absence of 7OTD, the approximately 23-kb WGA product was not detected in 1% agarose electrophoresis since the column is not suitable for long-DNA purification. However, the G4-forming regions and non-G4-forming regions were amplified from the purified WGA products by PCR; thus, the products amplified by WGA in the presence of 7OTD were analysed by PCR (Fig. 4). Although the non-G4-forming regions were amplified by PCR, only slight PCR amplification of the G4-forming regions was detected. Quantitative analysis of the band intensity revealed that the average WGA efficiencies in the non-G4-forming and G4-forming regions were 50% and 17%, respectively. These results demonstrate that the efficiency of amplification of the G4-forming regions by WGA was lower than that of the non-G4-forming regions in the presence of 7OTD.

### High-throughput sequencing of the WGA products for identification of G4 clusters in human genomic DNA

To identify the G4-forming regions on the human genome, high-throughput sequencing of the WGA products was performed on an Illumina HiSeq X10 platform using the paired-end mode (150 bp x2). The WGA products were purified before PCR analysis, as described above. In contrast, the WGA products were directly used as templates for high-throughput sequencing, without any purification because DNA polymerase-based reactions would be inhibited on the G4-forming regions in the presence of 7OTD during the library preparation and sequencing reaction. High-throughput sequencing yielded 337 million reads (35 × depth of coverage) and 311 million reads (32 × depth of coverage) for 7OTD and control libraries, respectively.

**Figure 5:**
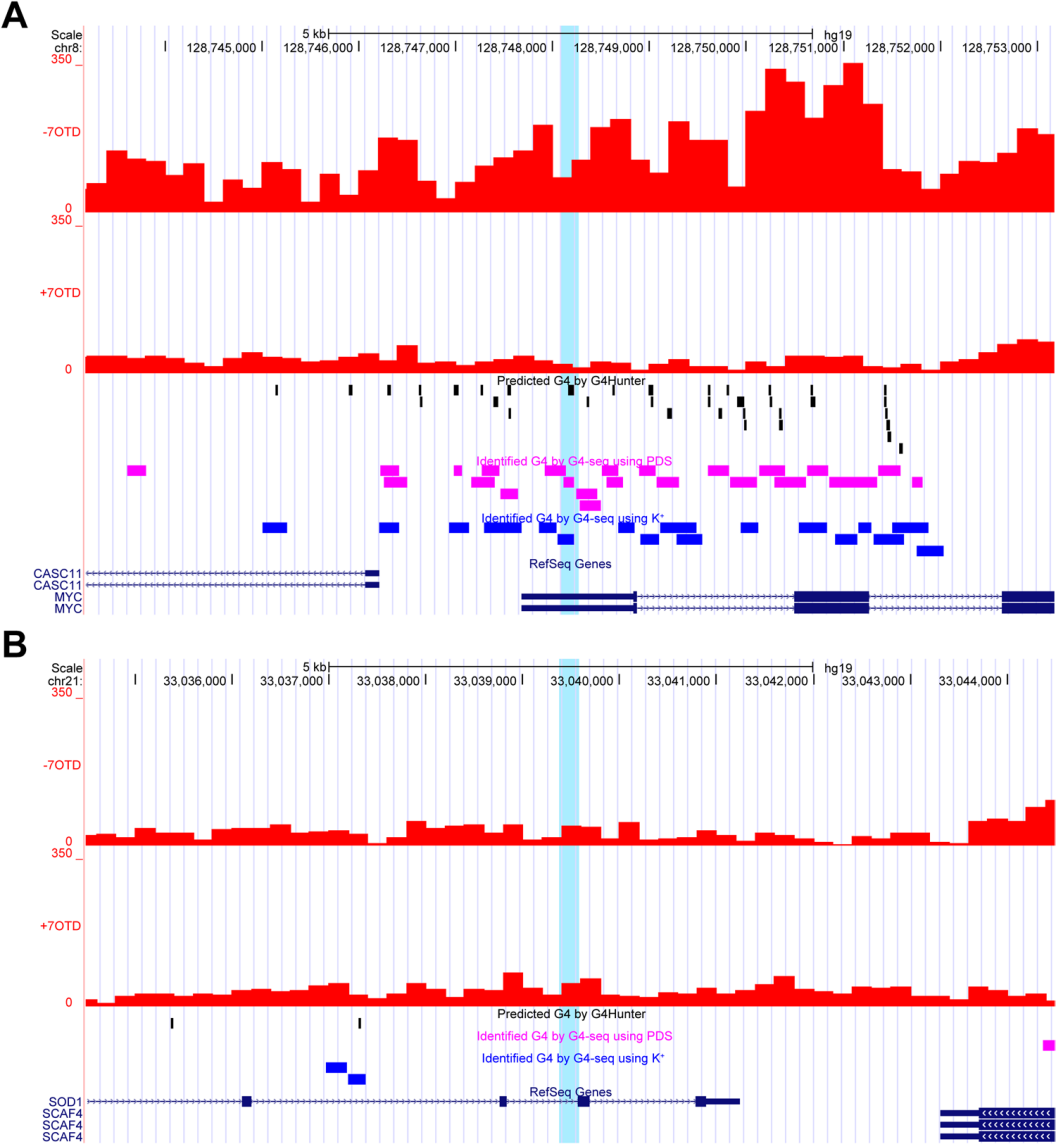
High-throughput sequencing of the WGA products. Sequencing results in *c-MYC* (**A**) and *SOD1* regions (**B**). The counted mapped reads are shown in red bars for the control library and the 7OTD library. G4-forming sequences identified by G4-seq using G4 ligand pyridostatin (PDS) or K^+^ are shown as pink or blue boxes, respectively. PQS are shown as black boxes, and PCR target regions are highlighted.

First, the mapped reads were counted per 200 bp window, with a sliding size of 200 for the entire genome. Consistent with the PCR analysis of the WGA products, a decrease of the mapped reads in the 7OTD library was detected in *c-MYC*, *c-KIT*, *BCL2* and *VEGFA* G4 regions (Fig. 5 and Fig. S5). In contrast, specific peaks at the G4-forming sequences were not detected in the regions because the decrease of the mapped reads was detected over the whole region. The occurrence of PQS predicted by G4Hunter is 5.02 per 10 kbp in the human genome^17^. In contrast, 35, 15, 26 and 60 PQS were predicted in the 10 kbp region of *c-MYC*, *c-KIT*, *BCL2* and *VEGFA* G4, respectively. Clusters of G4-forming sequences that promote transcription and replication-dependent DNA damage induced by a G4 ligand have been identified by ChIP-Seq for the DNA damage marker γH2AX^22^. ChIP-Seq demonstrated that the γH2AX domains were enriched on chromosomes that have high PQS frequencies. Our sequencing results also demonstrated that the mean depth of coverage of the 7OTD library was lower than that of the control library on chromosomes 16, 17, 19, 20 and 22, which have high PQS frequencies (Fig. S6). These results indicated that clusters of G4-forming sequences would be identified by counting the mapped reads over large windows in our sequencing results.

We then counted the mapped reads per 1.0, 2.5, 5.0 and 10 kbp windows with sliding sizes of 1.0, 2.5, 5.0 and 10 kbp, respectively. The correlation coefficients between PQS frequencies and ratios of the reads for the 7OTD library to the reads for the control library in 1.0, 2.5, 5.0 and 10 kbp windows were −0.52, −0.61, −0.65 and −0.66, respectively. Therefore, counted mapped reads per 10 kbp windows were utilised to identify G4 clusters (Supplementary Dataset 1). On the 25 γH2AX-enriched genes, the average PQS numbers was 24 per 10 kbp, the average G4 numbers identified by G4-seq using PDS was 9.8 per 10 kbp and the average ratio of the reads for the 7OTD library to the reads for the control library was 0.292 (Supplementary Dataset 2). In contrast, the average PQS numbers was 2.4 per 10 kbp, the average G4 numbers identified by G4-seq was 1.1 per 10 kbp and the average ratio of the reads was 1.92 on the two γH2AX-negative genes (Supplementary Dataset 3). Therefore, we defined the threshold for identification of G4 clusters as a PQS number is ≥24 per 10 kbp, the number of G4 identified by G4-seq using PDS is ≥10 and the ratio is ≤0.292. By these criteria, we identified 9,651 G4 clusters in the human genome (Supplementary Dataset 4). In the 9,651 G4 clusters, the average ratio of reads, number of PQS and number of G4 identified by G4-seq using PDS was 0.133, 39.8 and 14.9, respectively.

G4-seq using PDS identified 716,310 G4-forming sequences, whereas G4-seq using K^+^ identified 525,890 G4-forming sequences in human genomic DNA. This suggests that there are G4-forming sequences for which G4 folding is induced by a G4 ligand. G4 formations may be induced by G4 binding proteins in cells. Therefore, extraction of the ligand-inducible G4 clusters would be important. To extract G4 clusters, we used G4-seq results performed in K^+^-stabilised condition. The average number of G4 was 6.2 per 10 kbp on the 25 γH2AX-enriched genes. We defined the threshold for identification of the ligand-inducible G4 clusters from 9,651 G4 clusters as ≤6 G4 identified by G4-seq using K^+^. Using this criterion, we extracted 1,622 ligand-inducible G4 clusters (Supplementary Dataset 5).

Among identified 9,651 G4 clusters, 3,766 G4 clusters (39.0%) contained at least one transcriptional start site (Supplementary Dataset 6). In the entire genome, 25301 windows (8.3%) contain at least one transcriptional start site, indicating that G4 clusters are enriched for transcriptional start sites. It has been reported that ATRX interacts with PQS clusters to regulate gene expression^30^. These results, therefore, suggest that genes are regulated by G4 clusters rather than by one G4 structure.

## Discussion

ChIP-Seq for the DNA damage marker γH2AX is useful to identify G4 clusters that fold into G4 structures *in vivo*; however, the method was applied on only oncogenes and tumour suppressor genes because of the broad coverage of γH2AX signatures^22^. Moreover, G4 structure formation would be affected by the chromatin state *in vivo*. In contrast, we identified 9,651 G4 clusters in the whole human genome because our method directly detected G4 clusters that were stabilised by a G4 ligand *in vitro*. We also demonstrated that 3,766 G4 clusters contain at least one transcriptional start site. The phi29 DNA polymerase used in the WGA reaction is a replicative polymerase from the *Bacillus subtilis* phage phi29 (Φ29), which has strand displacement and processive synthesis properties^31^, meaning that it can imitate the replication process. G4-forming structures that play critical roles in replication have been identified, such as Rif1-binding sequences^32^. Therefore, the G4 clusters identified in this study would be involved in not only transcriptional regulation but also DNA replication.

Several structure-specific ligands that have the specificity to bind to different G4 structures have been reported; for example, 4,2-L2H2-6OTD and 5,1-L2H2-6OTD induced an antiparallel topology and a hybrid-type topology of telomeric DNA, respectively^33^. In addition, InEt2 and InPr2 G4 ligands specifically stabilised the parallel topology of *c-MYC*, *c-KIT1* and *c-KIT2* G4 structures^34^. The G4 ligand inhibited DNA polymerase extension on *c-MYC* G4 DNA, whereas no significant amount of stop product of telomeric G4 DNA was observed. Our method may thus contribute to the identification of G4 clusters containing specific G4 structures using topology-specific ligands. Moreover, the binding specificity of a telomestatin derivative against multimeric G4 structures can be improved by multimerization of the G4 ligand^35^. These results suggest that gene-specific G4 cluster ligands may be developed by multimerization of suitable G4 ligands, with designing the linker lengths.

It has been reported that the *Bcl-2* G4 structure and quadruplex structure of *C9orf72* repeat were stabilised by DNA methylation^36,37^. We reported that the initial elongation efficiency of PCR decreased with increasing DNA methylation levels in *VEGFA* and *RET* G4-forming sequences^38^, indicating that the G4 structures are also stabilised by DNA methylation. These reports suggest that our method could be applied to detect epigenetic modification by identifying G4 clusters stabilised by DNA methylation.

## Methods

### Analysis of inhibitory activity of 7OTD on PCR

Human genomic DNA was purified from HeLa (RBRC-RCB0007, RIKEN) cells using DNeasy blood and tissue kit (Qiagen). As G4-forming regions, *c-MYC*, c*-KIT*, *BCL2* and *VEGFA* G4-forming regions were used. As non-G4-forming regions, *MBD3L3*, *CD4*, *CNDP2*, and *SOD1* regions were used. All PCR primers (Table S1) were designed by Primer 3^39,40^. PCR was performed using 0, 1, 10, 100 or 1000 nM 7OTD, 500 nM each primer, 100 ng of human genomic DNA, 250 µM each dNTP and 0.5 U Ex Taq HS (Takara) with a buffer [25 mM TAPS (N-Tris(hydroxymethyl)methyl-3-aminopropanesulfonic acid), 2 mM MgCl_2_, 0.1 mM DTT, 5% DMSO (pH 9.3)] in a 20-µL solution. The thermocycling conditions were as follows: 95 °C for 5 min, followed by 35 cycles of 95 °C for 30 s, 59 °C for 30 s and 72 °C for 30 s. The PCR products were analysed by 2% agarose gel electrophoresis.

### Measurement of removal efficiency of BODIPY-labelled 7OTD from genomic DNA

HeLa genomic DNA (3.1 µg) and BODIPY-labelled 7OTD (10 µM) were mixed in a buffer [10 mM Tris–HCl, 100 mM KCl (pH 7.4)] in a 25-µL solution. After 10 min of incubation, 25-µL of 8 M LiCl was added and then incubated at 95 °C for 5 min. The BODIPY-labelled 7OTD was removed using Illustra MicroSpin G-25 Columns (GE), in accordance with the manufacturer’s protocol. After the purification, an approximately 50-µL sample was obtained. The fluorescence intensity of BODIPY-labelled 7OTD in this sample was measured by a microplate reader (Spark 10 M, Tecan).

### Analysis of effect of residual 7OTD on PCR

HeLa genomic DNA (3.1 µg) and 7OTD (1 µM) were mixed in a buffer [10 mM Tris–HCl, 100 mM KCl (pH 7.4)] in a 25-µL solution. After 10 min of incubation, 25-µL of 8 M LiCl was added and then 7OTD was removed, as described above. PCR was performed using 3.2-µL of the purified sample, 500 nM each primer, 250 µM each dNTP and 0.5 U Ex Taq HS (Takara) with a buffer [25 mM TAPS, 2 mM MgCl_2_, 0.1 mM DTT, 5% DMSO (pH 9.3)] in a 20-µL solution. The thermocycling conditions were as follows: 95 °C for 5 min, followed by 35 cycles of 95 °C for 30 s, 59 °C for 30 s and 72 °C for 30 s. The PCR products were analysed by 2% agarose gel electrophoresis. As a control, 1.6-µL of the mixture of genomic DNA and 7OTD was used as a template for PCR.

### Whole genome amplification in the presence of 7OTD

In the presence or absence of 1 µM 7OTD, WGA was performed using REPLI-g Mini Kit (QIAGEN), in accordance with the manufacturer’s protocol. Briefly, 2.5-µL of HeLa genomic DNA (100 ng) was incubated with 2.5-µL of denaturation solution at room temperature for 3 min and then 5-µL of neutralisation buffer was added. Next, 40-µL of master mix containing phi29 DNA polymerase with 7OTD was added and incubated at 30 °C for 16 h. To remove 7OTD, 25 µL of 8 M LiCl was added to 25-µL of the WGA product and then incubated at 95 °C for 5 min. The 7OTD was removed by LiCl and Illustra MicroSpin G-25 Columns (GE), as described above. The purified WGA product (3.2-µL) was used as a template for PCR in a 20-μL reaction volume, as described above.

### High-throughput sequencing of the WGA products

Sequencing libraries were prepared using the NEBNext Ultra II kit (NEB) and sequenced using the paired-end mode (150 bp x2) on the HiSeq X10 platform (Illumina) at Macrogen Inc. Obtained sequence reads were mapped onto the hg19 reference genome using BWA^41^. PCR-duplicate reads were removed using Picard^42^. In total, 311 and 337 million reads were obtained for the 7OTD and control libraries, respectively. Mapped reads per 0.2, 1.0, 2.5, 5.0 or 10 kbp windows with the sliding size of 0.2, 1.0, 2.5, 5.0 or 10 kbp were counted for the entire genome using bedtools 2.26.0, respectively^43^. The fold-change value and Fisher’s exact test p-value were calculated for mapped read counts per window. G-quadruplex forming sequences were predicted by the G4hunter program^17^, with default settings. The mapped reads per window, fold-change of mapped reads, and locations of predicted G4 quadruplex sites were visualised in.igv format on the Integrative Genome Viewer^44^ and on the USCS Genome Browser^45^.

## Electronic supplementary material

Supplementary Information

Dataset 1

Dataset 2

Dataset 3

Dataset 4

Dataset 5

Dataset 6

## References

[1] Hardin CC, Watson T, Corregan M, Bailey C (1992). Cation-dependent transition between the quadruplex and Watson-Crick hairpin forms of d(CGCG3GCG). Biochemistry.

[2] Sen D, Gilbert W (1988). Formation of parallel four-stranded complexes by guanine-rich motifs in DNA and its implications for meiosis. Nature.

[3] Sundquist WI, Klug A (1989). Telomeric DNA dimerizes by formation of guanine tetrads between hairpin loops. Nature.

[4] Eddy J, Maizels N (2006). Gene function correlates with potential for G4 DNA formation in the human genome. Nucleic Acids Res..

[5] Bochman ML, Paeschke K, Zakian VA (2012). DNA secondary structures: stability and function of G-quadruplex structures. Nat. Rev. Genet..

[6] Rhodes D, Lipps HJ (2015). G-quadruplexes and their regulatory roles in biology. Nucleic Acids Res..

[7] Verma A, Yadav VK, Basundra R, Kumar A, Chowdhury S (2009). Evidence of genome-wide G4 DNA-mediated gene expression in human cancer cells. Nucleic Acids Res..

[8] Catasti P, Chen X, Moyzis RK, Bradbury EM, Gupta G (1996). Structure-function correlations of the insulin-linked polymorphic region. J. Mol. Biol..

[9] Timmer C (2014). An isothermal titration and differential scanning calorimetry study of the G-quadruplex DNA-insulin interaction. J. Phys. Chem. B..

[10] Brooks TA, Kendrick S, Hurley LH (2010). Making sense of G-quadruplex and i-motif functions in oncogene promoters. FEBS J..

[11] Onel B (2016). A New G-Quadruplex with Hairpin Loop Immediately Upstream of the Human *BCL2* P1 Promoter Modulates Transcription. J. Am. Chem. Soc..

[12] Paeschke K, Capra JA, Zakian VA (2011). DNA replication through G-quadruplex motifs is promoted by the Saccharomyces cerevisiae Pif1 DNA helicase. Cell.

[13] Mani P, Yadav VK, Das SK, Chowdhury S (2009). Genome-wide analyses of recombination prone regions predict role of DNA structural motif in recombination. PLoS One.

[14] Ribeiro MM (2015). G-quadruplex formation enhances splicing efficiency of PAX9 intron 1. Hum. Genet..

[15] Huppert JL, Balasubramanian S (2005). Prevalence of quadruplexes in the human genome. Nucleic Acids Res..

[16] Eddy J, Maizels N (2008). Conserved elements with potential to form polymorphic G-quadruplex structures in the first intron of human genes. Nucleic Acids Res..

[17] Bedrat A, Lacroix L, Mergny JL (2016). Re-evaluation of G-quadruplex propensity with G4Hunter. Nucleic Acids Res..

[18] Cao K, Ryvkin P, Johnson FB (2012). Computational detection and analysis of sequences with duplex-derived interstrand G-quadruplex forming potential. Methods.

[19] Kudlicki AS (2016). G-quadruplexes involving both strands of genomic DNA are highly abundant and colocalize with functional sites in the human genome. PLoS One.

[20] Iida K (2013). Fluorescent-ligand-mediated screening of G-quadruplex structures using a DNA microarray. Angew. Chem. Int. Ed. Engl..

[21] Bay DH (2017). Identification of G-quadruplex structures that possess transcriptional regulating functions in the Dele and Cdc6 CpG islands. BMC Mol. Biol..

[22] Rodriguez R (2012). Small-molecule-induced DNA damage identifies alternative DNA structures in human genes. Nat. Chem. Biol..

[23] Chambers VS (2015). High-throughput sequencing of DNA G-quadruplex structures in the human genome. Nat. Biotechnol..

[24] Silander K, Saarela J (2008). Whole genome amplification with Phi29 DNA polymerase to enable genetic or genomic analysis of samples of low DNA yield. J. Methods Mol. Biol.

[25] Guo K (2007). Formation of pseudosymmetrical G-quadruplex and i-motif structures in the proximal promoter region of the *RET* oncogene. J. Am. Chem. Soc..

[26] Tera M (2009). Synthesis of a potent G-quadruplex-binding macrocyclic heptaoxazole. Chembiochem.

[27] Iida K, Nagasawa K (2013). Macrocyclic polyoxazoles as G-quadruplex ligands. Chem. Rec..

[28] Chung WJ (2013). Solution structure of an intramolecular (3 + 1) human telomeric G-quadruplex bound to a telomestatin derivative. J. Am. Chem. Soc..

[29] Woiczikowski PB, Kubar T, Gutiérrez R, Cuniberti G, Elstner M (2010). Structural stability versus conformational sampling in biomolecular systems: why is the charge transfer efficiency in G4-DNA better than in double-stranded DNA?. J. Chem. Phys..

[30] Law MJ (2010). ATR-X syndrome protein targets tandem repeats and influences allele-specific expression in a size-dependent manner. Cell.

[31] Blanco. L (1989). Highly efficient DNA synthesis by the phage phi 29 DNA polymerase. Symmetrical mode of DNA replication. J. Biol. Chem..

[32] Kanoh Y (2015). Rif1 binds to G quadruplexes and suppresses replication over long distances. Nat. Struct. Mol. Biol..

[33] Sakuma M (2016). Design and synthesis of unsymmetric macrocyclic hexaoxazole compounds with an ability to induce distinct G-quadruplex topologies in telomeric DNA. Org. Biomol. Chem..

[34] Diveshkumar KV (2016). Specific stabilization of *c-MYC* and *c-KIT* G ­quadruplex DNA structures by indolylmethyleneindanone scaffolds. Biochemistry.

[35] Abraham Punnoose J (2017). Adaptive and Specific Recognition of Telomeric G-Quadruplexes via Polyvalency Induced Unstacking of Binding Units. J Am Chem Soc.

[36] Lin J (2013). Stabilization of G-quadruplex DNA by C-5-methyl-cytosine in bcl-2 promoter: implications for epigenetic regulation. Biochem. Biophys. Res. Commun..

[37] Zamiri B, Mirceta M, Bomsztyk K, Macgregor RB, Pearson CE (2015). Quadruplex formation by both G-rich and C-rich DNA strands of the *C9orf72* (GGGGCC)8•(GGCCCC)8 repeat: effect of CpG methylation. Nucleic Acids Res..

[38] Yoshida W (2016). Detection of DNA methylation of G-quadruplex and i-motif-forming sequences by measuring the initial elongation efficiency of polymerase chain reaction. Anal. Chem..

[39] Koressaar T, Remm M (2007). Enhancements and modifications of primer design program Primer3. Bioinformatics.

[40] Untergasser A (2012). Primer3 - new capabilities and interfaces. Nucleic Acids Res..

[41] Li H, Durbin R (2009). Fast and accurate short read alignment with Burrows-Wheeler transform. Bioinformatics.

[42] Broad Institute of MIT and Harvard. Picard http://broadinstitute.github.io/picard/ (2017).

[43] Quinlan AR, Hall IM (2010). BEDTools: a flexible suite of utilities for comparing genomic features. Bioinformatics.

[44] Robinson JT (2011). Integrative genomics viewer. Nat. Biotechnol..

[45] Kent WJ (2002). The human genome browser at UCSC. Genome Res..

