# Supplementary material for: Identification of G-quadruplex clusters by high-throughput sequencing of whole-genome amplified products with a G-quadruplex ligand
Source: Sci Rep. 2018 Feb 15;8:3116. doi: 10.1038/s41598-018-21514-7 (PMC5814564; doi:10.1038/s41598-018-21514-7)
Supplement: Supplementary file 1 — Supplementary Information [file 41598_2018_21514_MOESM1_ESM.pdf]

Supplementary Information

Identification of G-quadruplex clusters by high-throughput sequencing of whole-genome amplified products with a G-quadruplex ligand

Wataru Yoshida[1,*], Hiroki Saikyo[1], Kazuhiko Nakabayashi[2], Hitomi Yoshioka[1], Daniyah Habiballah Bay[1,3], Keisuke Iida[4], Tomoko Kawai[2], Kenichiro Hata[2], Kazunori Ikebukuro[5], Kazuo Nagasawa[5] & Isao Karube[1]

[1]School of Bioscience and Biotechnology, Tokyo University of Technology, 1404-1 Katakura-machi, Hachioji, Tokyo 192-0982, Japan

[2] Department of Maternal-Fetal Biology, National Research Institute for Child Health and Development, 2-10-1 Ookura, Setagaya, Tokyo 157-0074, Japan

[3] Biology Department, Umm Al-Qura University, P.O. Box 715, Makkah, 21955, Saudi Arabia

[4] Molecular Chirality Research Center, Synthetic Organic Chemistry, Department of Chemistry, Graduate School of Science, Chiba University, 1-33 Yayoi, Inage, Chiba, 263-8522, Japan

[5] Department of Biotechnology and Life Science, Tokyo University of Agriculture and Technology, 2-24-16 Naka-cho, Koganei, Tokyo 184-8588, Japan

Correspondence and requests for materials should be addressed to:

W.Y. (email: yoshidawtr@stf.teu.ac.jp)

Table S1 List of primers sequences used in PCR.

Gene	Forward Primer (5'-3')	Reverse Primer (5'-3')	PCR product (bp)
c-MYC	TAGGCGCGCGTAGTTAATTC	CGGAGATTAGCGAGAGAGGA	191
c-KIT	AAAGAGCAGGGGCCAGAC	GGTCCACGTTCCAGCTCTC	192
BCL2	AAAGGATGACTGCTACGAAGTTCT	GTGACGTTACGCACAGGAAA	398
VEGFA	GTCGAGCTTCCCCTTCATT	CGCTACCAGCCGACTTTT	192
MBD3L3	AGATTGGGGTCACTGATTGG	GCACAGGGTTATTGTTACTGGA	213
CD4	AGCATTTCATAAGTCGCATGAT	AGGGCAATTCATACGCAAAA	222
CNDP2	CAGCACAGATGACTCGGATG	TGCACTATGTTCTGGCAAGG	194
SOD1	CCATCTTTCTTCCCAGAGCA	ACATTGCCCAAGTCTCCAAC	207

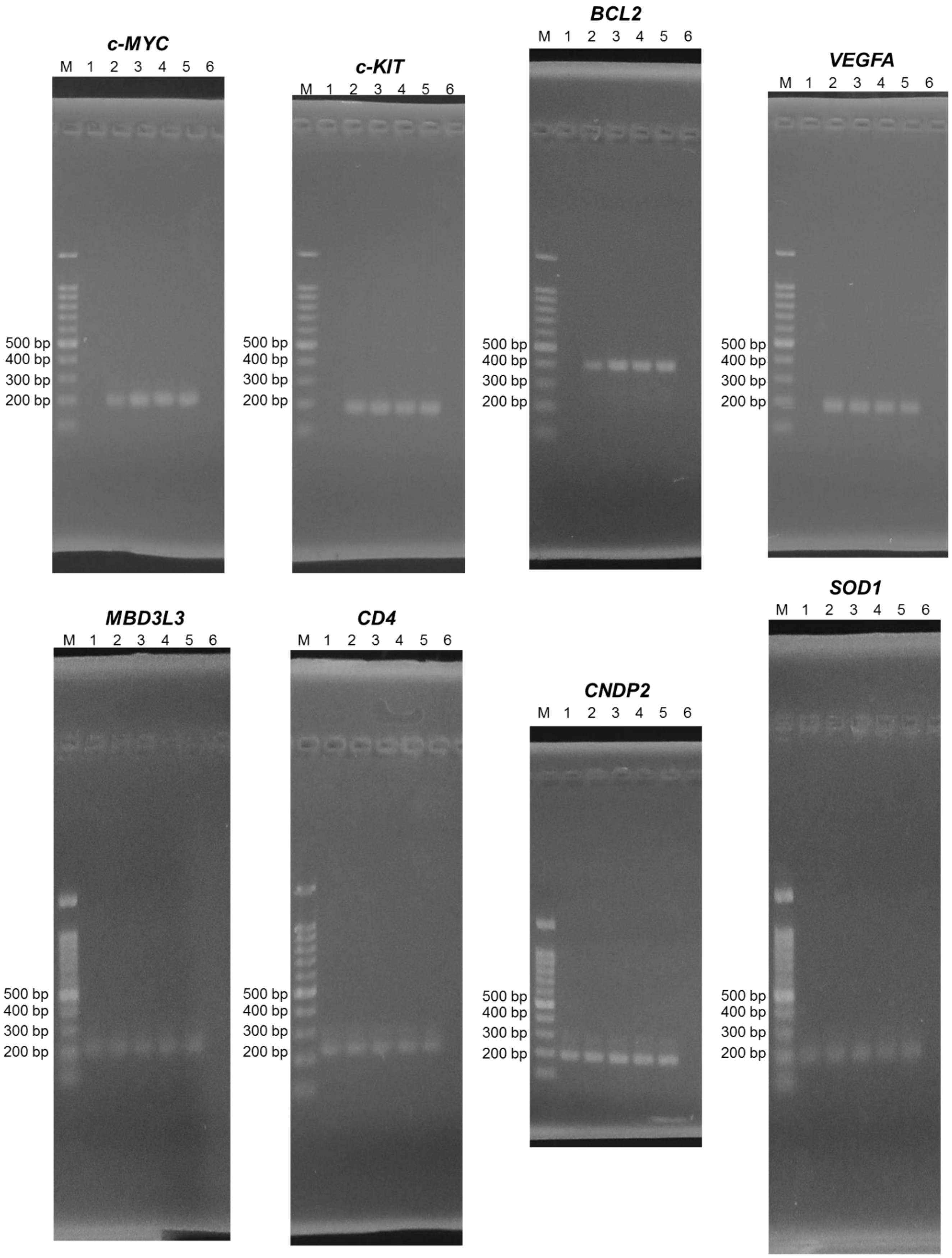

Fig. S1 Full length gels of Fig. 1.

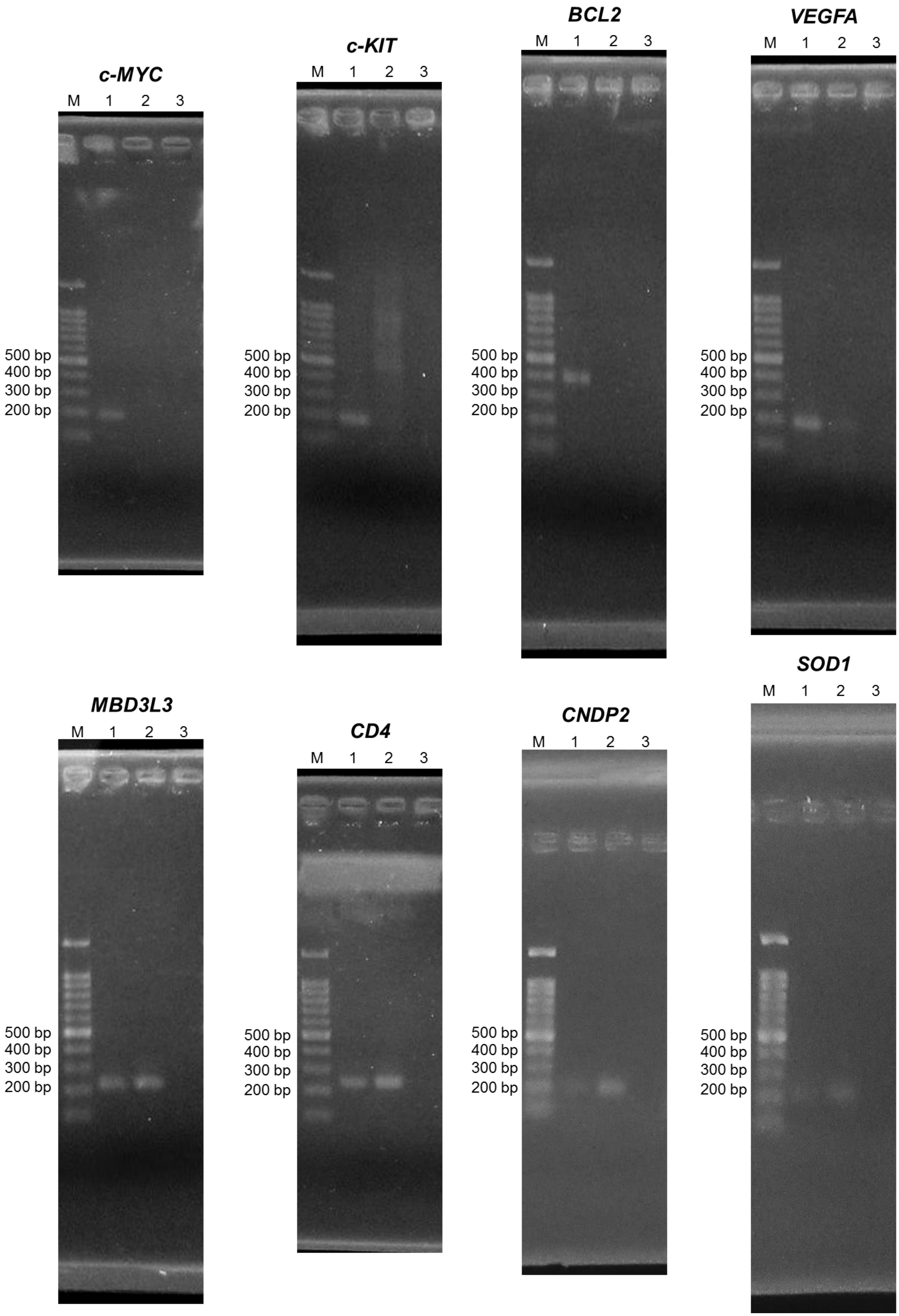

Fig. S2 Full length gels of Fig. 2.

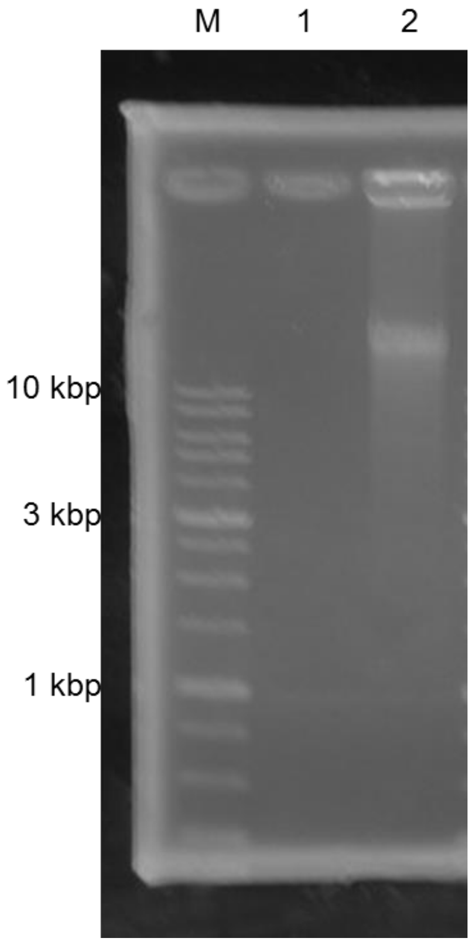

Fig. S3 Full length gels of Fig. 3.

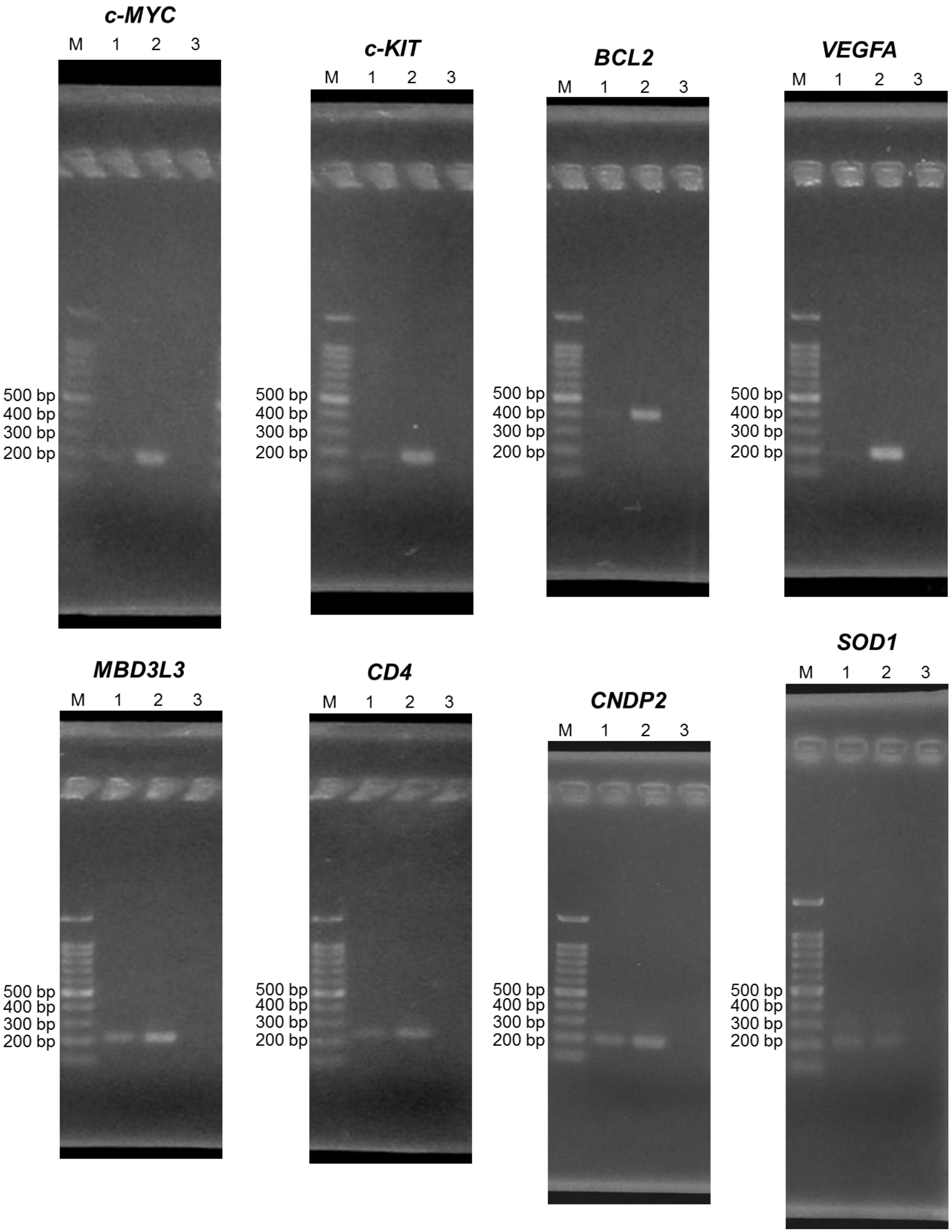

Fig. S4 Full length gels of Fig. 4.

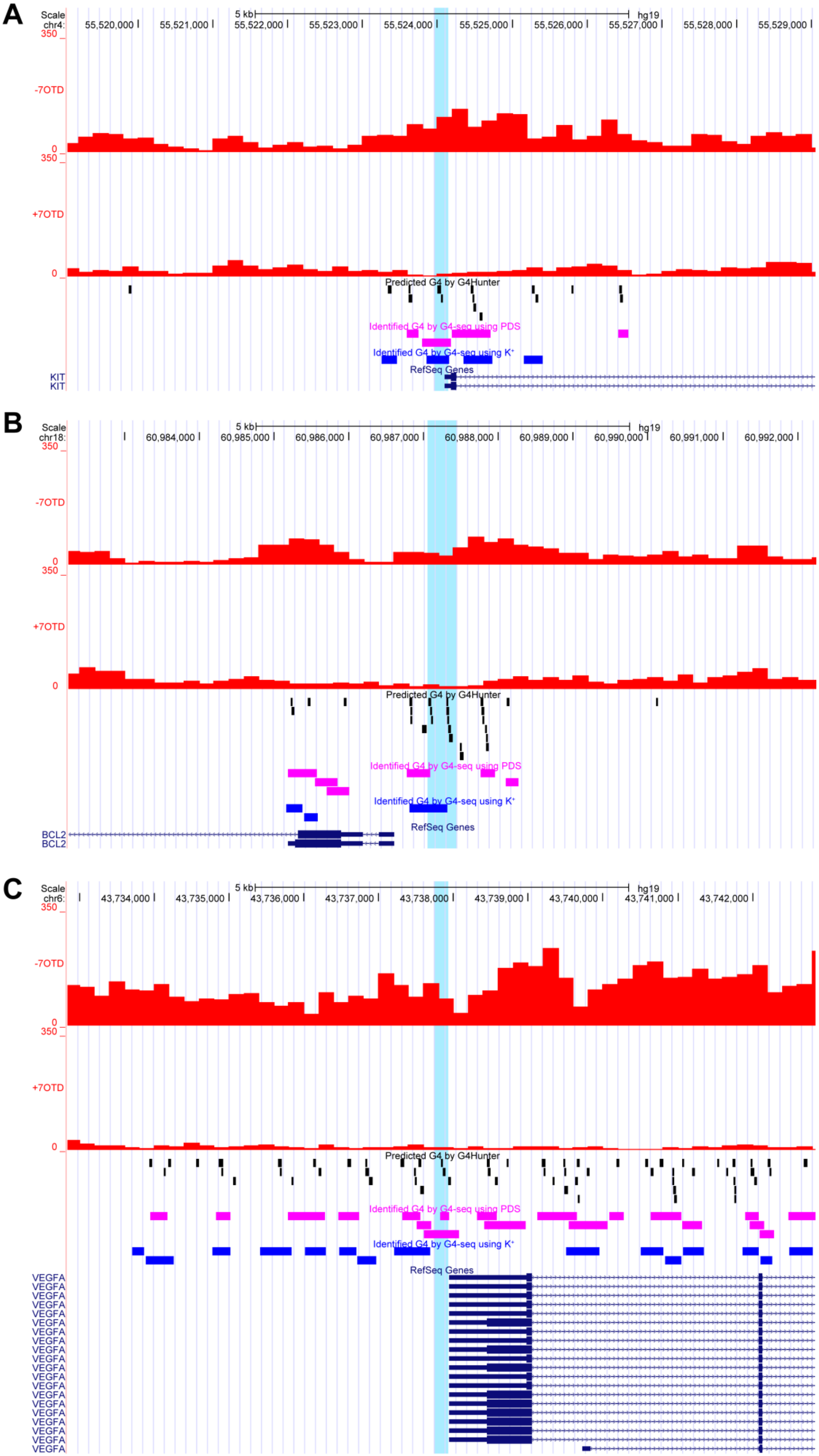
A
Scale
chr4:
5 kb
hg19
55,520,000
55,521,000
55,522,000
55,523,000
55,524,000
55,525,000
55,526,000
55,527,000
55,528,000
55,529,000
350
-7OTD
0
350
+7OTD
0
Predicted G4 by G4Hunter
Identified G4 by G4-seq using PDS
Identified G4 by G4-seq using K+
RefSeq Genes
KIT
KIT
B
Scale
chr18:
5 kb
hg19
60,984,000
60,985,000
60,986,000
60,987,000
60,988,000
60,989,000
60,990,000
60,991,000
60,992,000
350
-7OTD
0
350
+7OTD
0
Predicted G4 by G4Hunter
Identified G4 by G4-seq using PDS
Identified G4 by G4-seq using K+
RefSeq Genes
BCL2
BCL2
C
Scale
chr6:
5 kb
hg19
43,734,000
43,735,000
43,736,000
43,737,000
43,738,000
43,739,000
43,740,000
43,741,000
43,742,000
350
-7OTD
0
350
+7OTD
0
Predicted G4 by G4Hunter
Identified G4 by G4-seq using PDS
Identified G4 by G4-seq using K+
RefSeq Genes
VEGFA

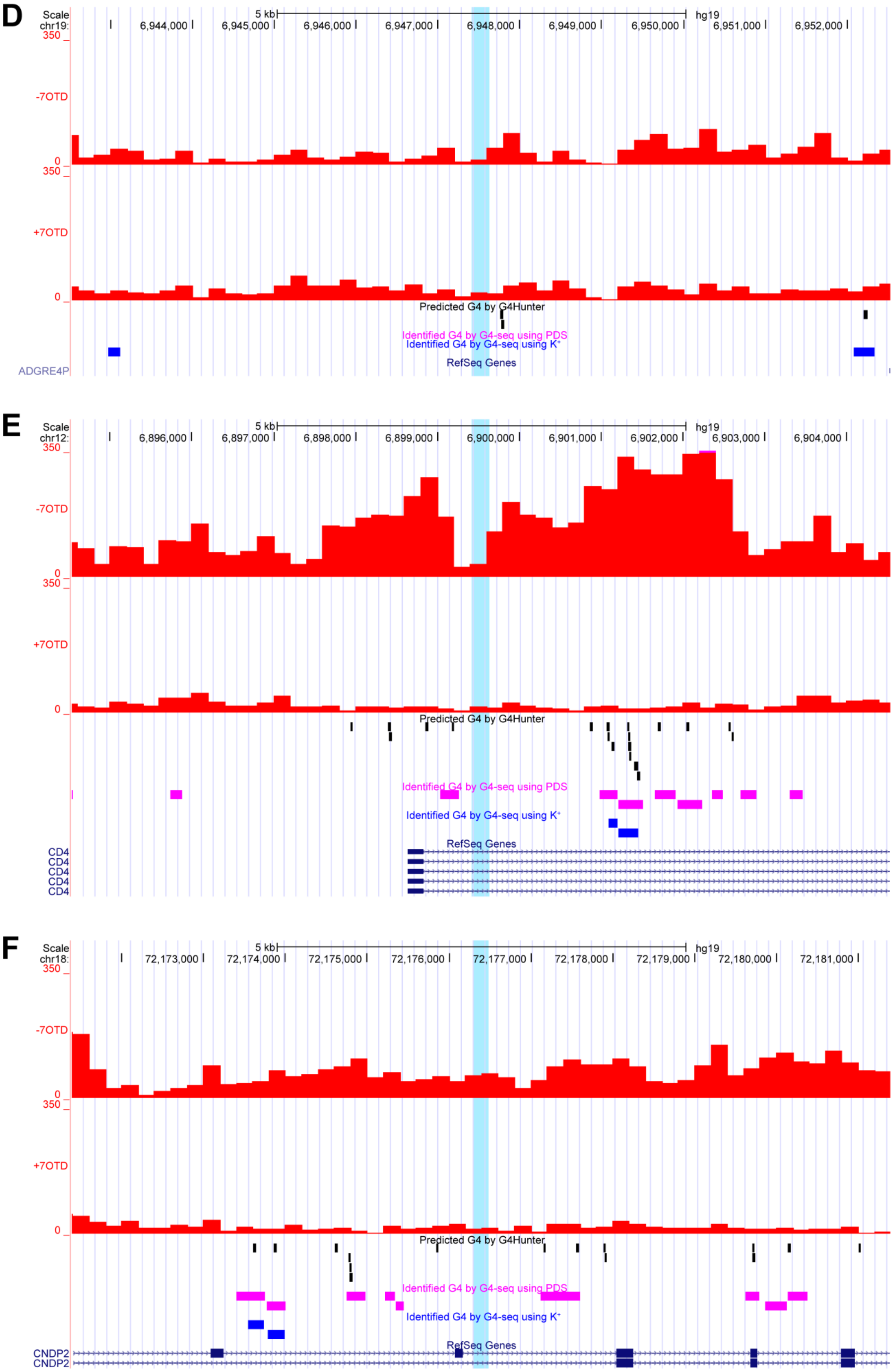

D
Scale
chr19:
5 kb
hg19
6,944,000
6,945,000
6,946,000
6,947,000
6,948,000
6,949,000
6,950,000
6,951,000
6,952,000
350
-7OTD
0
350
+7OTD
0
Predicted G4 by G4Hunter
Identified G4 by G4-seq using PDS
Identified G4 by G4-seq using K⁺
RefSeq Genes
ADGRE4P
E
Scale
chr12:
5 kb
hg19
6,896,000
6,897,000
6,898,000
6,899,000
6,900,000
6,901,000
6,902,000
6,903,000
6,904,000
350
-7OTD
0
350
+7OTD
0
Predicted G4 by G4Hunter
Identified G4 by G4-seq using PDS
Identified G4 by G4-seq using K⁺
RefSeq Genes
CD4
CD4
CD4
CD4
CD4
F
Scale
chr18:
5 kb
hg19
72,173,000
72,174,000
72,175,000
72,176,000
72,177,000
72,178,000
72,179,000
72,180,000
72,181,000
350
-7OTD
0
350
+7OTD
0
Predicted G4 by G4Hunter
Identified G4 by G4-seq using PDS
Identified G4 by G4-seq using K⁺
RefSeq Genes
CNDP2
CNDP2

Fig. S5 High-throughput sequencing of the WGA products. Sequencing results in *c-KIT* (A), *BCL2* (B), *VEGFA* (C), *MBD3L3* (D), *CD4* (E), and *CNDP2* (F). The counted mapped reads are shown in red bars for the control library and the 7OTD library. G4 identified by G4-seq with G4 ligand pyridostatin (PDS) or K^+ are shown as pink or blue boxes, respectively. PQS are shown as black boxes, and PCR target regions are highlighted.

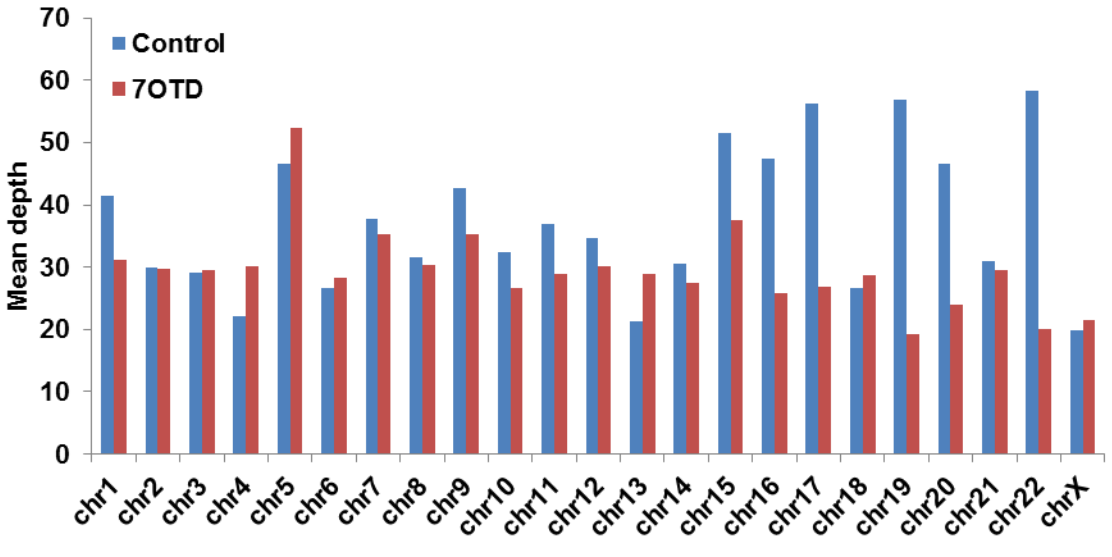

Fig. S6 The coverage depth of chromosome for the control library (blue) and the 7OTD library (red).
